# All-optical nonvolatile optical modulator for in-fiber operation

**DOI:** 10.1515/nanoph-2023-0212

**Published:** 2023-06-12

**Authors:** Zhihai Liu, Xiang Li, Siying Cheng, Yaru Li, Wei Jin, Yu Zhang, Yifan Qin, Yaxun Zhang, Shanshan Li, Andriy Lotnyk, Libo Yuan

**Affiliations:** Key Lab of In-Fiber Integrated Optics, Ministry Education of China, Harbin Engineering University, Harbin, 150001, P.R. China; Leibniz Institute of Surface Engineering, Permoserstr. 15, 04318 Leipzig, Germany; Laboratory of Infrared Materials and Devices, The Research Institute of Advanced Technologies, Ningbo University, Ningbo, Zhejiang 315211, P.R. China; College of Physics and Optoelectronic Engineering, Harbin Engineering University, Harbin, 150001, P.R. China; Photonics Research Center, Guilin University of Electronics Technology, Guilin, 541004, P.R. China

**Keywords:** all-optical modulation, integrated optics, optical fiber, phase-change materials

## Abstract

The control of information is a defining feature of the information age, and the optical modulator likewise has a crucial role in optical networks. The transmission, processing, and storage of data have demanded low energy consumption and high speed for photonic systems, promoting the development of electro-optic modulators to all-optical modulators. Although these all-optical modulation methods eliminate the photoelectric conversion, the disadvantage of volatile materials requiring continuous power supply when processing and retaining data in new materials-based devices increase energy consumption. We propose a Ge_2_Sb_2_Te_5_ (GST) integrated all-optical, nonvolatile optical modulator for in-fiber operation. The pulse-induced GST phase transition changes the reflectivity of the fiber end face, and this difference affects the result of the interference, achieving a modulation of output light intensity in interference spectra. The experimental results reveal that the device has obtained 13 dB interference intensity contrast in the telecommunications bands, and its response to a pump pulse is around 100 ns. Furthermore, we demonstrated the operation of the device as a scalar multiplication unit and a logic operation unit. The signal can be transmitted, processed, and stored in the fiber without photoelectric conversion. With the benefits of the switching power consumption of less than 100 nJ and the nonvolatile nature of GST, the device will be more energy-efficient in synchronous processing and storing. This in-fiber operating modulator lays the foundation for developing all-optical devices and networks.

## 1 Introduction

The development of optical communication technology in the information age puts higher requirements for all kinds of photonic integrated networks. Whether these systems are used for data transmission [1–3] or quantum computing [4, 5], they are eager to process more information with lower energy consumption and faster speed. As the fundamental component of the system, optical modulators play an essential role in many fields, even in photon computing, to solve the growing demand for information processing. To address the growing demand for information processing, optical modulators play an essential role in fields such as telecommunications networks and photonic computing [6–9]. All-optical devices are also indispensable in developing all-optical computers and photonic neural networks [10–12]. But the photoelectric conversion in traditional communication networks limit the synchronous processing and storing in the data transmission, increasing the overall energy consumption and complexity. It is necessary to develop an all-optical modulation device for efficient information networks.

In recent years, many studies on all-optical modulators have shown that researchers are interested in developing all-optical devices. On the one hand, from the aspect of whether materials can be driven by optical means, researchers search and create new materials to achieve all-optical modulation by light–matter interaction. Compared to conventional electro-optical modulation schemes achieved by exciting electro-absorption or electro-refractive index changes in semiconductor materials, new materials can break the bottleneck of electro-optical modulation schemes and overcome the disadvantage of weak electrogenic effect when the laser with communication wavelengths of 1310 nm and 1550 nm act on silicon materials [13, 14], and giving full play to the advantages of wide optical signal bandwidth and large capacity. For instance, with vanadium dioxide (VO_2_) as a metal–insulator transition material, the refractive index of VO_2_ can be significantly changed by laser [15]. Thus, a modulator based on laser-induced refractive index change can be constructed. However, the all-optical modulators based on VO_2_ are limited by the volatility of this material [16–18], so the devices often consume more energy in the process of transmitting, processing, and storing data.

Another direction that researchers focus on is how to combine materials with optical fibers better, so they have designed many schemes such as microfiber, microfiber ring, interlayer fiber, and interference structure to realize all-optical modulators [19–21]. Compared with electro-optical modulation systems, these all-fiber interconnection modulation schemes have the characteristics of anti-electromagnetic interference, high transmission rate, and low system weight. Moreover, simple combination and reliable interconnection between optical fiber devices are conducive to improving the density of the optical interconnection system. Despite the above advantages, the response time of these solutions is about milliseconds, and the manufacturing difficulty of complex structures also makes them unsuitable for large-scale development and application.

**Figure 1:**
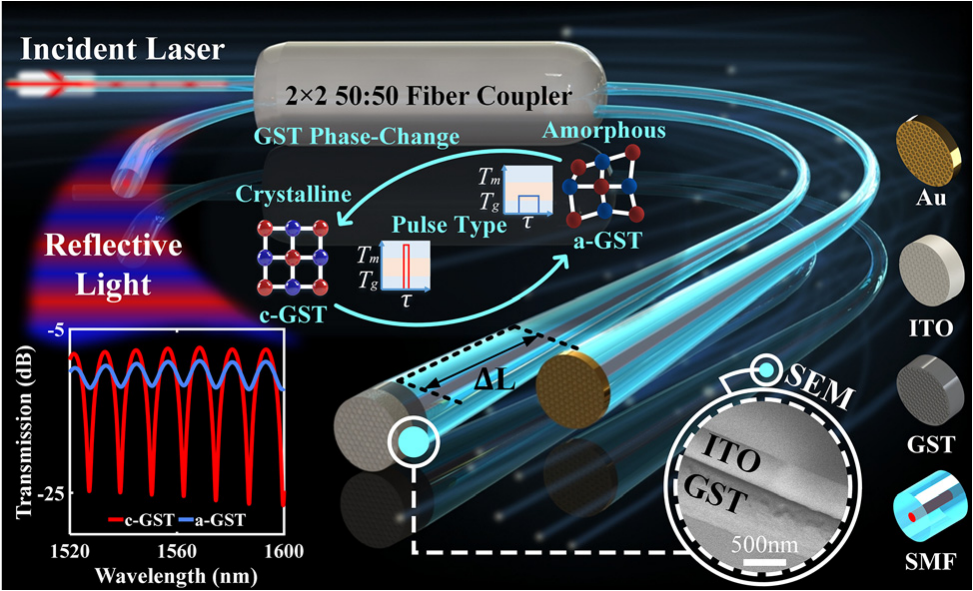
Schematic of the GST-integrated fiber optical modulator. The two fiber end faces on the right side of the fiber coupler are respectively coated with different materials as reflecting surfaces. One fiber on the left inputs signal light and control light, and the other fiber outputs reflected interference signals as the drop port, forming the optical fiber Michelson interferometer as a whole. The length difference Δ*L* between the two interference arms is ∼80 μm. One end of the fiber is coated with 500 nm GST and 200 nm ITO, and 10 nm gold film is sputtered on the other fiber end. (The middle illustration: when the atoms of GST are orderly distributed, it is shown as crystalline GST [c-GST], which can be switched to amorphous GST [a-GST] by high-power narrow-width pulse; On the contrary, the crystallization process can be completed by low-power wide-width pulse. Bottom right illustration: Scanning electron microscope [SEM] image of optical fiber end surface coated with GST and ITO film.)

We demonstrate an all-optical modulation scheme based on high refractive index contrast materials, which can meet the requirements of fast and low power consumption. This Ge_2_Sb_2_Te_5_ (GST) integrated nonvolatile all-optical modulator as sketched in Figure 1. The direct interaction between light–matter at the end face increases the feasibility of efficient all-optical modulation. The device is realized by separately growing GST and Au film at the end of the different interference arms of the optical fiber Michelson interferometer composed of a 2 × 2 coupler. The pump pulse sent into the structure can change the phase of GST on the end face, thus changing the reflectivity of the end face of the fiber. Therefore, the light intensity change in one interference arm transforms the extinction ratio of the entire interference spectrum. Finally, the intensity modulation of the signal light is realized through the pump laser pulse. In addition, we also study the scalar multiplication unit and logic operation unit using an optical modulator, which is expected to play a role in the low consumption and fast all-optical system.

## 2 Device fabrication

We integrate the GST into the Michelson interferometer composed of the fiber 2 × 2 coupler to realize the intensity modulation of the output light from the drop port of the interferometer. The structure of the device is shown in Figure 1. First, cut the fibers of the two output arms of the fiber coupler under the microscope to obtain two fibers with a length difference Δ*L* of ∼80 μm, and then clean the fiber tip with an arc to prepare for coating. After that, the optical fiber is placed in the sputtering chamber and when the working pressure in the cavity is lower than 5 × 10^−4^ Pa, high-purity argon (Ar) is filled. Finally, a GST film layer with a thickness of 500 nm is deposited through a magnetron radio-frequency (RF) sputtering system (CSWN-3200). Identically, we use the same system to sputter 200 nm indium tin oxides (ITO) films on the outer surface of the GST film as an anti-oxidation film by RF magnetron sputtering. So far, the GST-coated fiber end (GST-FE) has been prepared. Sputtering the thin film on the flat end face of the fiber helps to ensure better interaction between the GST and the transmitted laser in the fiber. The inset of Figure 1 shows the scanning electron microscope image of the GST-FE with ITO film. And the Au-coated fiber end (Au-FE) is fabricated by a similar method, the difference being that a 10 nm thick gold film is deposited using a direct current sputtering method to improve the essential reflectivity of this end face.

## 3 Working principles

Phase-change materials (PCMs) have become ideal for many all-optical devices due to their ability to reversible phase transition and nonvolatile data storage. Among them, the chalcogenide germanium antimony telluride has a significant functional feature, with high contrast in its optical properties between crystalline and amorphous phases [22–25]. For example, when at the wavelength of 1550 nm, the refractive index difference Δ*n* of GST between the two phases is 2.5, and the extinction coefficient difference Δ*k* is 1 [26], which makes it possible to achieve strong phase or intensity modulation by integrating materials in a small range. Meanwhile, The material properties of GST allow the transition between the crystalline and amorphous phases takes place in the nanosecond to subnanosecond [27, 28], and the GST has a high endurance of up to 10^15^ switching cycles [29], which allows the manufacturing of GST-integrated fast devices. The fact that GST can be converted to an amorphous state by heating it above the melting temperature (*T*_m_ (∼650 °C)) and quenching it, and that it can be re-crystallized by heating it to the glass-transition temperature (*T*_g_ (∼150 °C)) [30, 31]. This makes the laser pulse suitable as heat stimuli for optical operations. Moreover, benefiting from the nonvolatile nature of the GST, the final phase can remain stable for several years without continuous bias energy, making devices more energy-efficient [32–34].

**Figure 2:**
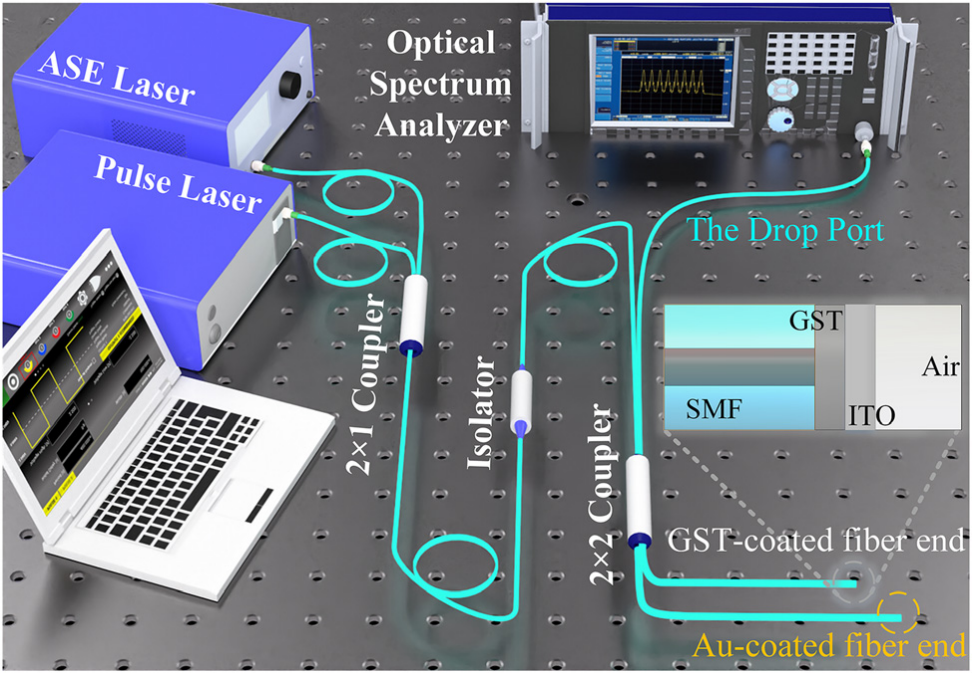
Measurement scheme of the optical system. The light from C + L-band amplified spontaneous emission (ASE) laser is used as a signal light, and the computer-controlled pulse laser sends different pulses as pump light, reversibly switches the phase of GST on the fiber end, and outputs different Michelson interference results to the optical spectrum analyzer through the drop port.

Therefore, the GST in chalcogenide PCMs has become the preferred material for constructing optical devices [35, 36]. And utilizing GST as high-index contrast material can provide strong modulation and nonvolatility for in-fiber optical modulators [37, 38]. In the experiment, selecting appropriate pulses to excite phase transition is a prerequisite for the operation of PCM-based devices [39]. The GST is usually heated above the *T*_m_ by high-power short pulses, followed by rapid cooling below the *T*_g_, resulting in the fixation of atoms due to a sharp decrease in mobility, which shows as long-range disordering, i.e., an amorphous phase; whereas by using intermedium-power long pulses to heat GST above the *T*_g_ for a period of time to induce nucleation, the material will transform into an ordered crystalline phase [40, 41]. The intensity modulation of the optical modulator can be realized by pulse laser induced amorphous to the crystalline phase transition of GST [42]. Thereby we constructed the optical circuit as shown in Figure 2. And we pump pulses with a pulse width of 100 ns to crystallize the GST, and conversely, in the amorphization operation, pulses with a pulse width of 10 ns are injected.

Figure 2 shows the experimental arrangement used to realize the device. In this case, the signal light (HY-ASE-C + L-N-17-BD-FA, CONNECT) and the control light are sent to the second coupler from two ports of the first 2 × 1 coupler, where the number of control pulses emitted by the pump pulse laser (1550 nm, VFLS-1550-M-DTS-1-FA, CONNECT) is controlled by a pulse generator (Pulse Rider PG-1072, Active Technology). In the Michelson interferometer composed of the 2 × 2 coupler, the signal light is first split into two with an equal power ratio and sent into the two interference arms from the input arm of the Michelson interferometer.

The GST phase transition induced by optical pulses changes its refractive index, which allows the GST-coated fiber to be used as an all-optical modulator to modulate the intensity of light transmitted in it by changing the reflectivity of the end face. In our study, the reflectivity of Au-FE is around 65 % at C + L-band. And when GST is in the initially amorphous phase with a low refractive index, GST-FE exhibits low reflectivity. Thus, the light intensity difference in the two interference arms of the Michelson interferometer is significant, making the interference extinction ratio (ER) of interference more minor. The interfering input signal light will then output from the drop port of the Michelson interferometer. However, when GST is transformed into a crystalline phase, its refractive index increases significantly, which makes the reflectivity of GST-FE close to that of Au-FE. Then the output interference light in the drop port has a large ER. This result has also been verified by simulation.

**Figure 3:**
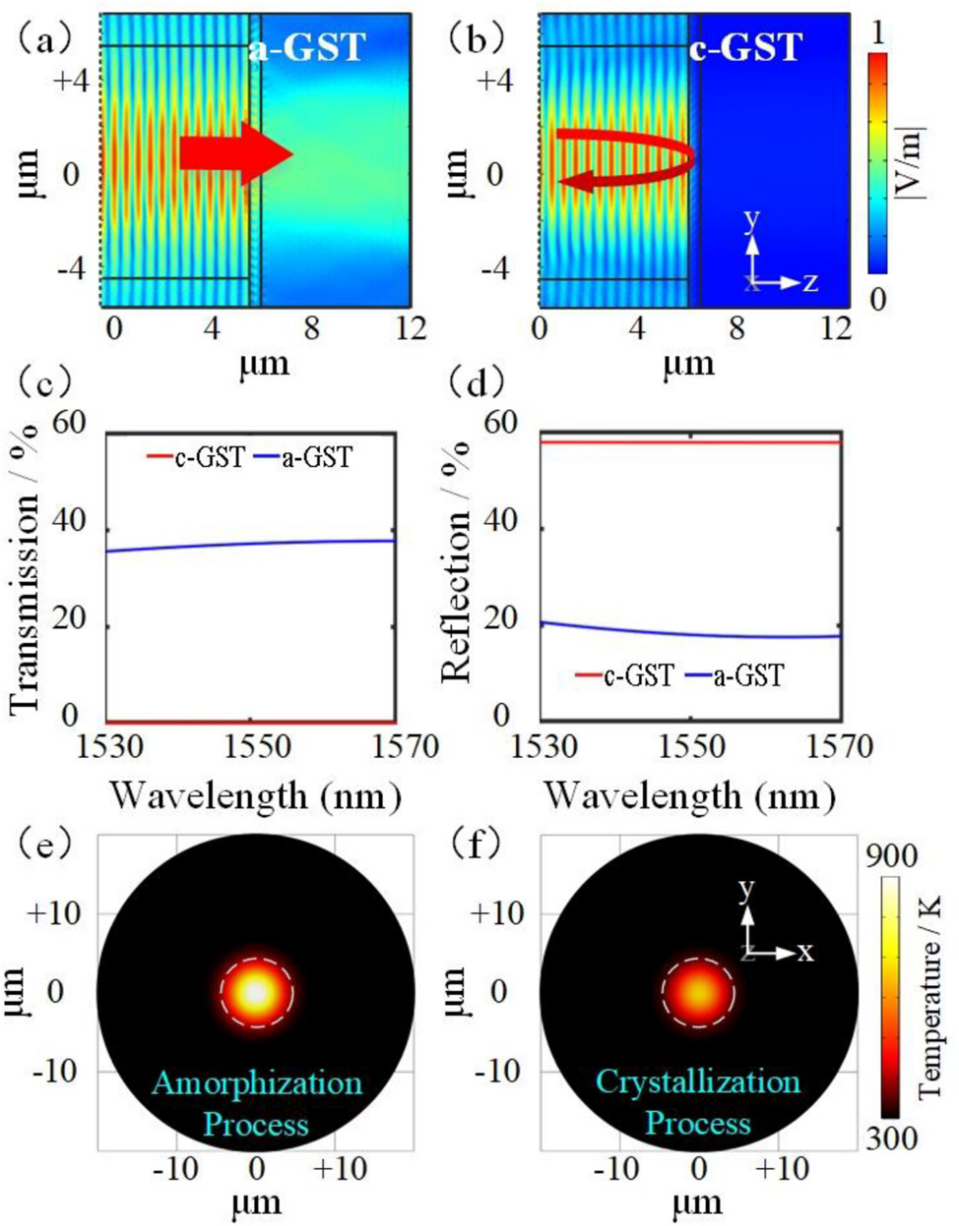
Simulation results of the film structure. (a) Simulation results of light distribution on fiber end face when GST is in the amorphous phase. (b) Simulation results of light distribution on fiber end face when GST is c-GST. (c) Transmittance *T*_r_ at the fiber and materials interface when GST is in different phases. (d) Reflectance *R*_e_ at the interface when GST is in different phases. (e) Temperature distribution on the GST film after the amorphization pulse. (f) Temperature distribution on the GST film after the crystallization pulse.

We simulate the distribution of the outgoing light field of the single fiber end face coated with GST of different phases. The results are shown in Figure 3(a) and (b). They show that when GST is in the amorphous phase, the signal light is emitted directly, while the signal light is reflected back to the fiber when GST is in the crystalline phase. Meanwhile, using GST and ITO as double-layer films, the optical transmission characteristics of the fiber (SiO_2_) and the film structure interface are calculated when the laser is incident from the fiber through the film into the air. The reflectivity *R*_e_ and transmittance *T*_r_ of the interface in different phase states are shown in Figure 3(c) and (d), and the absorbance *A*_b_ in both cases can be obtained according to Equation: *A*_b_ + *T*_r_ + *R*_e_ = 1. The reflectivity of the interface at 1550 nm was measured to be 47 % (c-GST) and 14 % (a-GST) for the different GST phases, respectively. This is due to the manufacturing defect of the film structure [43], resulting in slight deviations between the experimental and ideal simulation results. These results show that the crystalline state of GST exhibits higher reflectivity than the amorphous state based on the thermal parameters in reference [44]. We simulated the temperature distribution of this film structure under the action of different laser pulses by the finite-element method. The laser pulses acted as a heat source on the GST surface within the diameter range of 9 μm of the fiber core, and the high-power short pulses heated the GST to the *T*_m_, while the low-power long pulses only brought the GST to the *T*_g_.

The GST with different phases on the end face of the optical fiber makes the end face have different reflectivity. This difference in reflectivity only results in different reflected light intensity, avoiding the drift of the interference spectrum caused by the change of OPD. Therefore, in the experiment, the contribution of our optical fiber modulator to the intensity modulation mainly comes from the intensity change at the interference valley. The results are shown in the illustration in Figure 1, the output light intensity of the drop port varies with the phase state of GST, and there is a maximum light intensity contrast ratio at the interference valley in the spectrum. Here, the light intensity contrast ratio is defined as the maximum difference of output optical power at the same wavelength when GST is in different phase states; put another way, that is, when the optical modulator is in the ‘ON’ or ‘OFF’ state.

## 4 Measurement results

**Figure 4:**
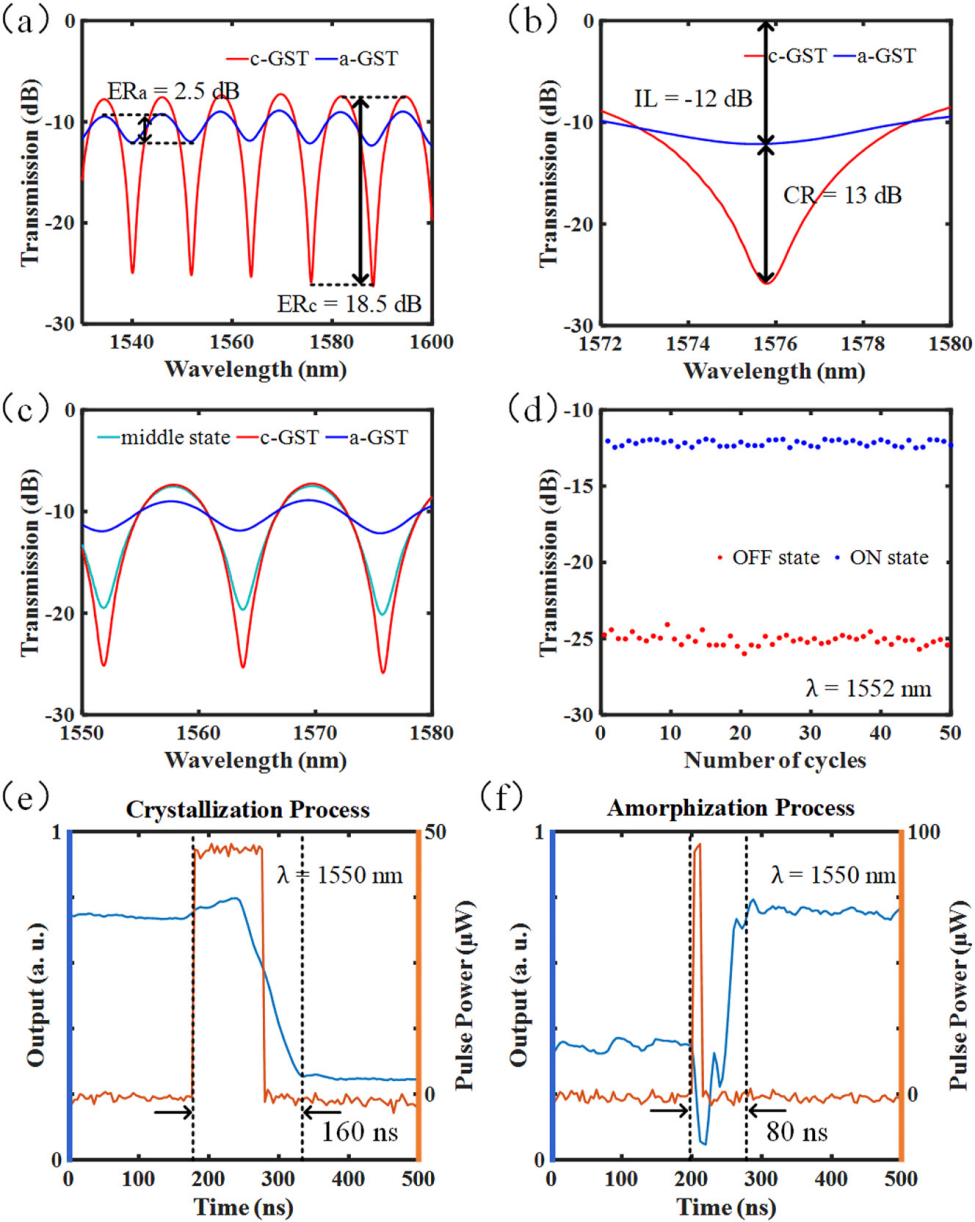
Experimental results of intensity modulation of the optical modulator. (a) The output spectra of the device in the C + L band and their respective extinction ratios (ER) when GST is switched to different phase states. (b) At an interference valley of the interference spectrum, the insertion loss of the device and the contrast of the output intensity under different states. (c) Control the number of crystallization pump pulses to output intermediate light intensity. (d) Repeatability test results of the device in 50 cycles. (e) Response time of the device to 100 ns pump pulse during crystallization. (f) Response time of the device to 10 ns pump pulse during amorphization.

Different excitation means determine different GST-phase states. In the experiment, the initial state of the GST in the prepared GST-FE is amorphous (a-GST), then the transmission spectrum is shown in the blue curve in Figure 4(a). The extinction ratio ER_a_ of the initial spectrum is 2.5 dB. After that, ten laser pulses with energy of 55 nJ are pumped to the GST-FE to realize the crystallization of GST. The repetition frequency of these 100 ns pulses is 1 kHz. The measured spectrum in the case of crystalline phase GST (c-GST) is shown in the red curve in Figure 4(a), and the extinction ratio ER_c_ of this interference spectrum reaches 18.5 dB. For the re-amorphous operation, rapid annealing is needed to make the GST phase-change, and the cooling rate of the material after the pulse action increases significantly with the decreasing of the pump pulse width, so we send 5 × 10 ns high-energy pulses, where the energy of each pulse is 93 nJ with the same repetition frequency of 1 kHz. Such an operation can make the GST return to the initial state; this means that the light intensity can be switched between the ‘ON’ and ‘OFF’ states to realize the intensity modulation of the transmitted light. The insertion loss of the optical intensity modulator and the light intensity contrast ratio of the device in the two states are −12 dB and 13 dB at 1576 nm, respectively, as shown in Figure 4(b).

In addition, in the process of pumping crystallization pulse heating GST, controlling the amount of total energy can make the material in the mixed-phase condition. GST can be stable in the intermediate state between the crystalline and amorphous phases. Note that the intermediate state here is a mixed state consisting of crystalline and amorphous regions, and this mixed state exhibits optical transmission properties that are intermediate between high reflectivity (c-GST) and low reflectivity (a-GST) [44], and this mixed state of the GST also has long stability without heat stimuli. Figure 4(c) shows that different pumping methods of the control laser can put the device in other states. The intermediate state in the figure is achieved by sending five crystallization pulses of 100 ns. Also, we test the device state in multiple cycles, and each cycle includes a crystallization and re-amorphous operation. The results in Figure 4(d) verify the repeatability of the device. In fact, due to GST properties, its switching cycle is up to 10^15^ times, and it can stay in the final state for many years.

Additionally, the dynamic response of the optical modulator to the pump pulse is also studied. In this case, the monochromatic light input device as the signal light and then connected to the photodetector from the output. After pumping crystallization and amorphization pulses, the measured dynamic response curve is shown in Figure 4(e) and (f). The device’s response time to the crystallization and amorphization pulses is 160 ns and 80 ns, respectively. It may be possible to achieve sub-nanosecond or even shorter device switching speeds by optimizing pulse parameters, such as using picosecond pulses. Note that, due to the nonvolatility of the GST, no more energy is required to hold the final state after switching, which makes the device more energy-efficient than volatile material-based modulators during continuous operation.

## 5 Discussion

The above results prove that this GST-integrated optical fiber modulator can realize the modulation function of high optical intensity contrast ratio through all-optical operation and has nonvolatile in the bidirectional reversible switching operation. The availability of such an in-fiber all-optical modulation technique opens up the possibility of low-power all-optical networks. Furthermore, the development of modulators for solving complex calculations makes it more necessary to develop optical modulators that use all-optical means to realize in-fiber operations, such as conventional calculations and logic operations.

**Figure 5:**
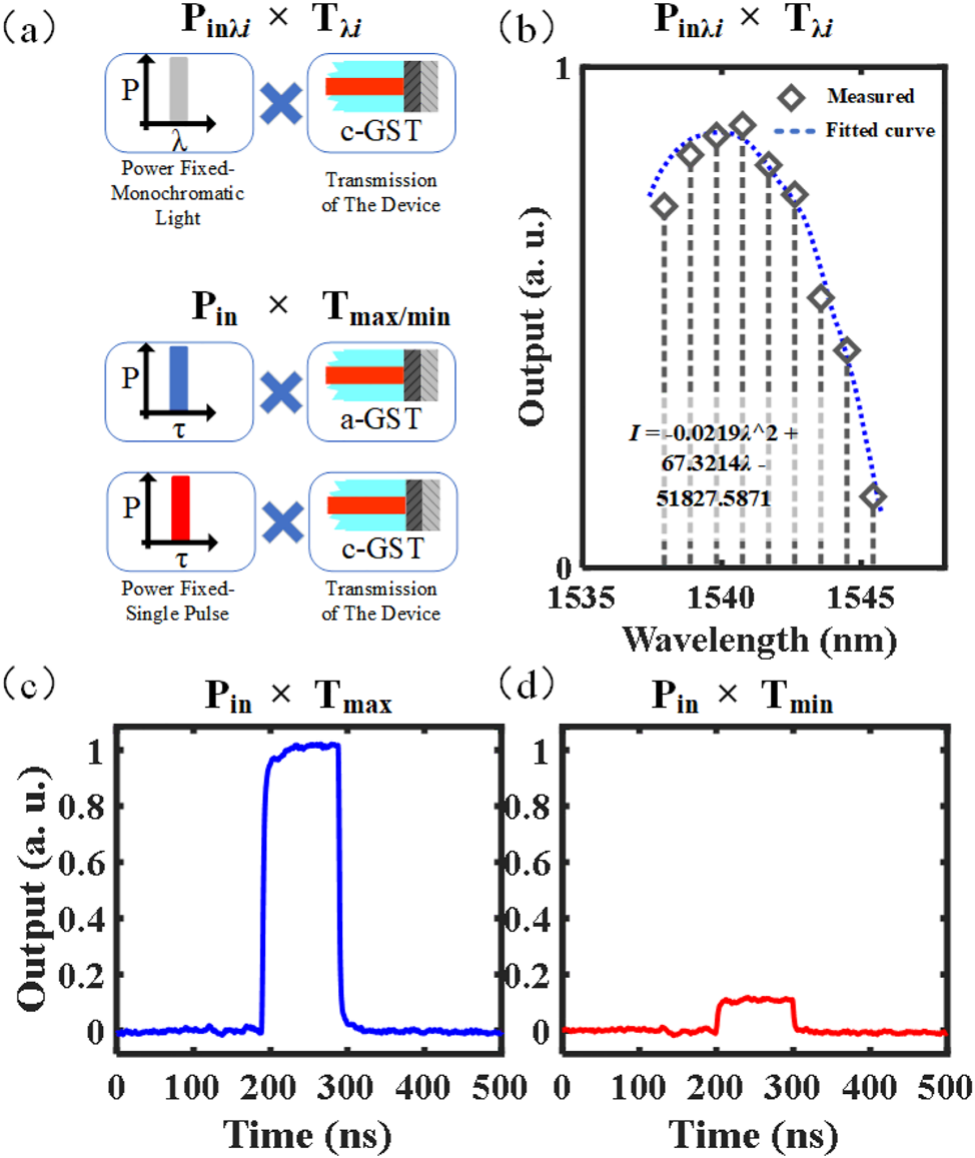
Scalar multiplication scheme of GST-integrated optical modulator. (a) Multiplication scheme of scalar *P*_in_ and *T* in two working modes. (Upper figure: *P*_inλ*i*_ represents the power of monochromatic light at different wavelengths, and *T*_λ*i*_ represents the transmittance of the device to varying wavelengths under a c-GST situation; lower figure, *P*_in_ represents the same single-pulse power, *T*_max_ and *T*_min_ represent the transmittance under a-GST and c-GST situation, respectively. The *P*_inλ*i*_ and *P*_in_ are both low-energy transmission signal lights with fixed power and do not cause the phase-change of GST.) (b) The first scheme measures the product of scalar *P*_inλ*i*_ and *T*_λ*i*_ at the output, where the transmittance *T*_λ*i*_ changes gradually with the wavelength. (c) and (d) The second scheme measures the product of scalar *P*_in_ and *T*, and the output is modulated by the maximum or minimum value of transmittance *T*.

We demonstrate the multiplication of two scalars, ‘*P*’ and ‘*T*,’ using a GST-integrated optical modulator as the basic unit. Where ‘*P*’ represents the input laser power, where the power is less than the pump power that will cause GST phase-change; ‘*T*’ represents the unit’s transmission modulated by the pump pulse The two operation modes of the multiplier are shown in Figure 5(a): The above figure shows the multiplication of scalar *P*_inλ*i*_ and *T*_λ*i*_. In this case, the GST in the device is always in the c-GST state, and the transmittance *T*_λ*i*_ at different wavelengths is different, resulting in different outputs after the monochromatic light of the same power, but different wavelengths is input; However, in the operation mode shown below, the transmission *T* is controlled by the pump pulse to be at the maximum (*T*_max_) or minimum (*T*_min_). Then a single pulse with the same power input will output the maximum or minimum pulse. As demonstrated, the measured result after multiplying two scalars is shown in Figure 5(b)–(d). Figure 5(b) shows that the product can be distributed between the maximum and minimum in this case, while Figure 5(b) and (c) show that the device can output the product of input signal *P*_in_ and *T*_max_ or *T*_min_. For instance, *T*_min_ represents that the device’s transmission is the lowest in the c-GST state. Therefore, the product in Figure 5(d) is almost zero. The multiplication processing of signals is based on all-optical operations, and after direct interaction between light and matter, the output pulse can be directly used as the calculation result.

**Figure 6:**
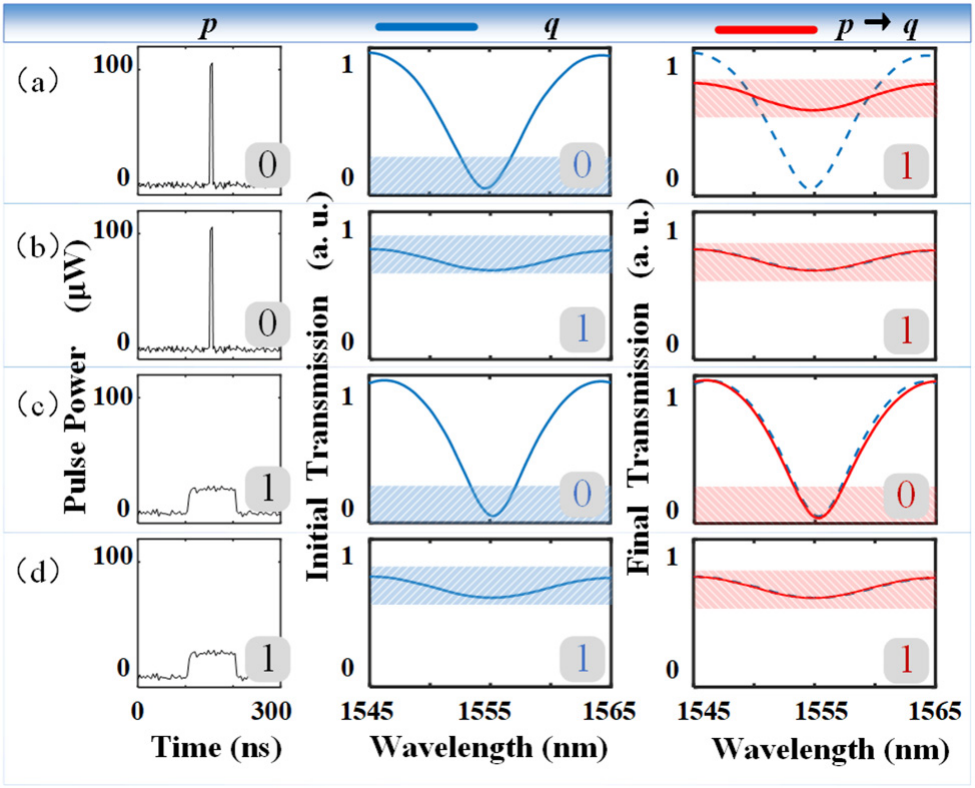
Logical operation scheme of GST-integrated optical modulator. (a)–(d) Correspond to four cases where the logic input *p* and *q* take different values, where ‘*p*’ refers to the applied pulse, when the pulse width is 10 ns, *p* = 0, and when the pulse width is 100 ns, *p* = 1; and ‘*q*’ represents the transmittance of the device in the initial state, *q* = 0 when the transmittance is low, and vice versa; The final state of the device is the logical operation result of *p*-IMP-*q*, which is defined as 0 at low transmittance and 1 at high transmittance.

In addition, we use this nonvolatile optical modulator for one fundamental logic operation. We demonstrate that when there are two variables, ‘*p*’ and ‘*q*,’ the device can be used to operate the material implication (IMP). That is, the logic operation result of *p*-IMP-*q* is equivalent to that of (NOT*p*)OR*q*. The truth table of this logic operation is shown in the figure at the lower right corner of each curve result in Figure 6(a)–(d). In the experiment, we used logic variables p and q to represent the pulse parameters applied, and the initial transmission of the optical modulator, respectively. We defined the narrow pulse as ‘*p* = 0’ (The high energy 10-ns pulse which is enough to induce the phase transition of GST), the wide pulse with low energy as ‘*p* = 1’ (The pulse width is 100 ns, but its energy is lower and it’s insufficient to induce the phase transition). And it is defined that when GST is in the crystalline phase, the device’s output at the interference valley is minimal, ‘*q* = 0’. On the contrary, when the output intensity of the device caused by amorphous GST is extremely high, which means ‘*q* = 1’. Finally, the result of logic operation *p*-IMP-*q* is determined by the final transmission of the output interference spectrum. When the light intensity at the interference valley is maximum, it means *p*-IMP-*q* = 1; and when it is minimal, it means *p*-IMP-*q* = 0. Taking Figure 6(a) as an example, the narrow-width pump pulse on the left represents *p* as 0, and the spectral in the middle shows that the minimum initial transmission of the device represents *q* as 0. Then, the spectral change on the right shows that the output transmission increases; *p*-IMP-*q* is 1. As a result, when such an all-optical device integrated with the fiber is added to the appropriate system, it can meet the working requirements as a logical computing device.

## 6 Conclusions

In summary, we have demonstrated the function of the GST-integrated all-optical modulator, which provides for nonvolatile optical intensity modulation near the C and L telecommunications bands. The modulation behavior is realized by depositing GST on the end face of one interference arm of the Michelson interferometer. Adjusting the phase state of GST can change the end-face reflectivity, thus causing the extinction ratio of interference spectrum change. Interference valley wavelengths can be set, anywhere within the transparency range of the GST, by adjusting the path difference of the interference arms. In this work, the device provides 13 dB optical intensity contrast at the interference valley of the above wave band, and the device’s response time to a single pump pulse is in the nanosecond scale and its switching energy is also at the nanojoule level. Then we demonstrated the scalar multiplication unit and logic operation unit based on the device. The all-optical operation in these schemes is nonvolatile, which means that the fiber-integrated device can combine transmitting, processing, and storing signals without continuous bias energy and faster speed, without the intervention of a photoelectric converter. Meanwhile, these functions form the basis of the signal processing system, realize the in-fiber processing of the transmitted optical signal, and endow the fiber with the ability beyond transmission and sensing technology. This work provides technical and experience support for optical memory, optical calculator, optical system, and optical network.
